# Long Noncoding RNAs as Orchestrators of CD4^+^ T-Cell Fate

**DOI:** 10.3389/fcell.2022.831215

**Published:** 2022-06-20

**Authors:** Chang Liu, Yanli Zhang, Zhanchuan Ma, Huanfa Yi

**Affiliations:** ^1^ Central Laboratory, The First Hospital of Jilin University, Changchun, China; ^2^ Key Laboratory of Organ Regeneration and Transplantation, Ministry of Education, Changchun, China

**Keywords:** long noncoding RNA, plasticity, CD4^+^ T-cell differentiation, immune modulation, immune diseases

## Abstract

CD4^+^ T cells differentiate towards different subpopulations through the regulation of lineage-specific cytokines and transcription factors, which flexibly respond to various immune challenges. However, considerable work has demonstrated that the CD4^+^ T-cell differentiation mechanism is complex and not limited to transcription factors and cytokines. Long noncoding RNAs (lncRNAs) are RNA molecules with lengths exceeding 200 base pairs that regulate various biological processes and genes. LncRNAs have been found to conciliate the plasticity of CD4^+^ T-cell differentiation. Then, we focused on lncRNAs involved in CD4^+^ T-cell differentiation and enlisted some molecular thought into the plasticity and functional heterogeneity of CD4^+^ T cells. Furthermore, elucidating how lncRNAs modulate CD4^+^ T-cell differentiation in disparate immune diseases may provide a basis for the pathological mechanism of immune-mediated diseases.

## Introduction

CD4^+^ T cells possess vital properties in immunomodulation, mediating adaptive immune responses by differentiating into various subpopulations, such as T-helper 1 (Th1), Th2, Th17, Th9, and Th22 cells; regulatory T cells (Tregs); and T follicular helper (Tfh) cells. Th1 cells function to destroy endocellular viruses and bacteria, whereas Th2 cells target extracellular pathogens and are necessary to fight parasitic infections (Saravia et al., 2019). Similar to Th2 cells, Th9 cells are involved in fighting parasitic infections and inducing allergic diseases (Sehra et al., 2015). Additionally, Th17 cells stimulate protective immunity against extracellular bacteria and fungi, and Th22 cells express specific interleukin (IL)-22 subsets and are distinguished from Th17 cells, which also produce IL-22 (Saravia et al., 2019). Tregs figure prominently in maintaining the status of immune balance and preventing autoimmune diseases and transplant rejection. Tfh cells, another subgroup of CD4^+^ T cells, activate B cells and regulate humoral immune responses synergistically, and are a crucial class of T cells in germinal centers (Crotty, 2019; Niogret et al., 2021).

Long noncoding RNAs (lncRNAs) are noncoding RNA molecules with lengths of greater than 200 base pairs and a structure similar to that of mRNA, although without the ability to encode protein. Importantly, lncRNAs are characterized by cell-specific than protein-coding genes and are involved in multiple biological processes such as regulating epigenetic inheritance, cell cycle, and cell differentiation (Cabili et al., 2011). Moreover, lncRNAs exert their various functions via a variety of mechanisms, including regulation of transcription through chromatin, modulation of protein activity, direct participation in post-transcriptional regulation of mRNA, and regulation of microRNAs (miRNAs) through sponging (Thomson and Dinger, 2016; Akhade et al., 2017). Abnormal lncRNA expression can influence the emergence, development, and prognosis of various immune diseases by controlling CD4^+^ T-cell differentiation. Thus, further analyses of lncRNAs may facilitate the elucidation of the molecular mechanisms of differentiation plasticity in CD4^+^ T cells.

Here, we review the functional plasticity of CD4^+^ T-cell subsets and how their effector function is influenced by the transcriptional regulatory network triggered by T cells, which includes lncRNAs. Comprehending the modulation mechanisms and identifying the participants in CD4^+^ T-cell plasticity may raise our awareness of the pathogenesis of immune-related diseases and facilitate the identification of novel therapeutic targets.

## LncRNAs Facilitate the Lineage Shift Between Th1 and Th2 Cells

Naïve CD4^+^ T cells are stimulated to differentiate into Th1 cells mainly by the cytokines interferon (IFN)-γ and IL-12. Signal transducer and activator of transcription (STAT) 1 is activated by IFN-γ signaling, which stimulates the production of T-bet, a transcription factor of the Th1 lineage. IL-12 further drives T-bet expression through STAT4. T-bet enhances the Th1 phenotype by promoting IFN-γ and IL-12 expression (Zhang et al., 2014). Furthermore, Th2 cells can be induced to differentiate by IL-4 and IL-2. IL-4 accelerates *IL-4* transcription and the production of the transcription factors musculoaponeurotic fibrosarcoma (c-MAF) and GATA3 by activating STAT6. Among these factors, c-MAF promotes IL-4 expression and shuts down IFN-γ generation, whereas GATA3 makes Th2-cell differentiation primarily by stimulating the induction of IL-5 and IL-13. IL-2 induces Th2 cytokine production by activating STAT5 (Zhu, 2015). The Th1/Th2 balance is critical for the workings of the immune system, and when that balance is disrupted in favor of either side, disease can occur.

The lncRNA transcript *Tmevpg1*, also called *NeST* or *Ifng*-AS1, was originally discovered in the context of Theiler’s virus infection, and the researchers found that mice without *Tmevpg1* expression could not control intracranial viral infection (Collier et al., 2012). *Tmevpg1*, an essential gene involved in specific expression of the IFN-γ gene (*Ifng*), is located near and co-expressed with *Ifng* (Collier et al., 2012). Several studies have suggested that transcription of *Tmevpg1* is to rely on STAT4 and T-bet (Collier et al., 2012; Collier et al., 2014). Mechanistically, the epigenetic remodeling of *Tmevpg1* is induced by T-bet, resulting in the aggregation of transcription factors NF-κB and Ets-1 at this site (Collier et al., 2014). Furthermore, T-bet completed specific expression of *Ifng* in Th1 lineage by epigenetic remodeling of Tmevpg1 specific enhancers and *Ifng* specific enhancers. Thus, *Tmevpg1* transcription is Th1 selective. *GATA3-AS1* is a divergent lncRNA contiguous to *GATA3* that has been shown to be essential for efficient transcription of *IL-5* and *IL-13* genes and for *GATA3* transcription under a Th2 bias. *GATA3-AS1* forms permissive chromatin markers at the *GATA3-AS1-GATA3* locus via H3K27 acetylation and H3K4 di-/trimethylation. In addition, *GATA3-AS1* binds the MLL methyltransferase component to form an R-loop at the *GATA3/GATA3-AS1* locus, thereby leading MLL methyltransferase to the gene sites (Gibbons et al., 2018). Accordingly, the lncRNA *GATA3-AS1* promotes Th2 lineage development through *SATA3*. Moreover, lincR-*Ccr2*-5′AS regulates the expression of chemokine in mice, including *Ccr1*, *Ccr3*, *Ccr2*, and *Ccr5*. LincR-*Ccr2*-5′AS has been shown to be regulated by GATA-3 and, lincR-*Ccr2*-5′AS together with GATA-3, regulates Th2 cell-specific gene expression and is critical for Th2 cell migration (Hu et al., 2013).

Locus control region (LCR) plays a major role in the regulation of Th2 cytokine genes (Koh et al., 2010). In humans, the gene encoding an lncRNA cluster overlaps the *RAD50* gene (encoding a double-strand break repair protein) and is adjacent to the TH2 LCR in mice, so it is named *TH2-LCR* lncRNA (Spurlock et al., 2015). The *TH2-LCR* lncRNA cluster consists of four transcripts, all of which are Th2-lineage specific and co-expressed with *IL-4*, *IL-5*, and *IL-13* but not *RAD50* (Spurlock et al., 2015). One function of *TH2-LCR* lncRNA is to promote transcription of *IL-4* and *IL-13* by promoting the formation of chromatin marker H3K4Me3. However, the mechanism of *IL-5* transcription is still unclear (Spurlock et al., 2015). *TH2-LCR* is necessary for Th2 cytokine production and positively regulates Th2 subset formation. Additionally, chromatin-related lncRNA linc-*MAF-4* targeting the Th1 subpopulation is inversely correlated with the expression of c-MAF, a Th2-associated transcription factor encoded by the *MAF* gene. Linc-*MAF-4* negatively regulates *MAF* transcription by using chromatin modifier LSD1 and Zeste homolog 2 (EZH2) to regulate the enzyme activity of EZH2 on the *MAF* promoter (Ranzani et al., 2015). Accordingly, linc-*MAF-4* may suppress Th2-cell differentiation.

The lncRNA *AW112010* potentiates inflammatory T-cell differentiation, which exhibits high expression in Th1 and Th17 cells and low expression in Th2 cells. Studies have shown that *AW112010* interacts with the histone demethylase KDM5A to reduce H3K4me3 at the *IL-10* gene locus, inhibit IL-10 expression, and promote inflammatory T-cell differentiation (Yang et al., 2020). Collectively, *Tmevpg1*, *GATA3-AS1* and *Th2-LCR* are examples of lncRNAs that regulate the expression of adjacent protein-coding genes. These genes are frequently co-expressed with their corresponding lncRNAs. By contrast, the lncRNA lincR-*Ccr2-5*′*AS* affects the expression of adjacent genes, demonstrating that some lncRNAs can also facilitate the transcription of adjacent related genes rather than just being limited to co-expression. LncRNAs can also participate in transcriptional regulation through chromatin, including histone modification, DNA methylation, and chromatin remodeling. As described above, linc-*MAF-4* recruits the chromatin modifiers to regulate *MAF* transcription, *GATA3-AS1* recruits target gene sites through histone modification enzymes, and *AW112010* inhibits IL-10 expression by decreasing H3K4me3 at the *IL-10* gene locus. These findings indicate that lncRNA modulates the open or closed state of chromatin, which is determined by epigenetic factors.

The lncRNA nuclear paraspeckle assembly transcript 1 (*NEAT1*) is an important component of paraspeckles, a substructure that is widely found in mammalian nuclei (Fox and Lamond, 2010). Paraspeckles can regulate gene expression, and *NEAT1* is the skeleton that determines whether paraspeckles can form (Clemson et al., 2009). Previous studies have shown that *NEAT1* is up-regulated in many cancers, and its high expression often leads to poor prognosis of patients (Chakravarty et al., 2014; Chen et al., 2015; Ma et al., 2016). *NEAT1* also plays a role in CD4^+^ T-cell differentiation. Studies have shown that *NEAT1* can enhance the level of STAT6 by repressing STAT6 ubiquitination and degradation, thereby increasing Th2-cell proliferation (Huang et al., 2021). Systemic lupus erythematosus (SLE) is a systemic autoimmune disease. The transformation of Th1/Th2 cell balance to Th2 is considered to be one of the key processes in the pathogenesis of SLE (Marder, 2019). Notably, *NEAT1* is highly expressed in peripheral blood mononuclear cells (PBMCs) of patients with SLE and negatively correlated with the Th1/Th2 ratio, thus influencing the progression of systemic lupus erythematosus (Jiang et al., 2021). The mechanism of action of *NEAT1* reflects another function of lncRNAs, which bind to specific proteins and regulate protein activity. This transcriptional regulation may involve either a *cis* or *trans* mechanism, and the target proteins are usually transcription factors (Long et al., 2017).

The lncRNA plasmacytoma variant translocation 1 (*PVT1*) is encoded by *PVT1* gene, and is usually highly expressed in various malignant tumor tissues or cells, while the expression level is low in normal tissues (Jin et al., 2019). *PVT1* is a potential predictor of cancer progression and patient prognosis (Jin et al., 2019). LncRNA *PVT1* has been shown to function in the differentiation of CD4^+^ T cells through miRNAs. In many patients with asthma, chronic airway inflammation is caused by Th2 cells (Hammad and Lambrecht, 2021). In ozone-induced exacerbation of asthma, *PVT1* activates the asthma-related phosphatidylinositol 3-kinase (PI3K)/AKT/mammalian target of rapamycin (mTOR) pathway by inhibiting *miR-15a-5p*, leading to a decrease in Th1/Th2 ratio, thereby promoting the progression of asthma. Specifically, IL-4, IL-10, and GATA3 proteins levels increased, whereas IFN-γ, IL-2, and T-bet levels decreased (Wei et al., 2020). *PVT1* has also been shown to exhibit activity in other pathways. Depletion of *PVT1* in Th1 cells results in reduced IFN-γ secretion and Th1 cell dysfunction. This process also reduces the glycolysis gene transcription factor Myc expression and inhibits cell proliferation (Fu et al., 2020). Sjögren’s syndrome (SS) is a chronic inflammatory autoimmune disease involving exocrine glands (Fox, 2005). Excessive proliferation and activation of CD4^+^ T cells in salivary gland tissue is a hallmark of SS (Fu et al., 2020). LncRNA *PVT1* upregulation maintained Myc expression in CD4^+^ T cells of SS patients, while inhibition of glycolysis attenuated SS-like autoimmune response (Fu et al., 2020). *PVT1* may be a potential therapeutic target for SS. LncRNA growth arrest specific 5 (*GAS5*) is a well-known tumor suppressor (Gao et al., 2017). According to report, *GAS5* is associated with T-cell differentiation in immune-related diseases (Song et al., 2021). Allergic rhinitis (AR) is a well-documented allergic disease mediated by Th2 cells (Ji et al., 2021). The lncRNA *GAS5* is overexpressed in nasal mucus and nasal mucosal epithelial cells of AR patients. This lncRNA may inhibit Th1-cell differentiation by downregulating the expression of EZH2 and T-bet, and promote the differentiation of CD4^+^ T cells into Th2. LncRNA *GAS5* destroys the balance of Th1/Th2, leading to the occurrence of AR (Zhu et al., 2020a). Another study explored the mechanisms through which *GAS5* enhances Th2-cell differentiation in AR, and found that *GAS5* and *CircHIPK3* induced GATA-3 upregulation by regulating the common target *miR-495*, promote Th2-cell differentiation and aggravate AR (Zhu et al., 2020b). LncRNA *GAS5* may be a potential biomarker for the development and progression of AR. In distinct clinical contexts, both *PVT1* and *GAS5* play roles in Th1- and Th2-cell development. As a result, we speculate that the same lncRNAs may perform distinct or comparable activities in different settings by mediating multiple pathways.

The lncRNA metastasis-associated lung adenocarcinoma transcript 1 (*MALAT1*) is found in early non-small-cell lung cancer (NSCLC) and can predict metastasis and patient prognosis in early NSCLC (Li et al., 2018). In bronchial asthma, lncRNA *MALAT1* inhibits the expression of *miR-155* in CD4^+^ T cells through sponging and acts in conjunction with the *CTLA-4* gene to alter T-cell activity. As a result, the Th1/Th2 balance is biased toward the Th2 lineage, thereby promoting the development of bronchial asthma (Liang and Tang, 2020). LncRNA taurine upregulated 1 (*TUG1*) is one of the earliest lncRNAs found to be associated with human disease. It is abnormally expressed in many malignant tumors and is involved in the development of many cancers and cardiovascular diseases (Guo et al., 2020). A recent study found the role of lncRNA *TUG1* in promoting Th2-cell differentiation in asthma. LncRNA *TUG1* and B7-H3 were upregulated in peripheral blood of asthmatic children, while *miR-29c* was downregulated. The activity of B7-H3 in Th2-cell differentiation was inhibited by *miR-29c*. LncRNA *TUG1* regulates the expression of B7-H3 through sponge *miR-29c* and, thus promoting Th2-cell differentiation (Sun et al., 2021). This suggests that lncRNA *TUG1* may be involved in the acute attack of asthma. To summarize, the lncRNAs *PVT1*, *GAS5*, *MALAT1*, and *TUG1* can bind with corresponding miRNAs to regulate Th-cell differentiation, highlighting the roles of lncRNAs in the regulation of miRNAs. Indeed, some lncRNAs carry sequences complementary to certain miRNA sequences, enabling them to bind miRNAs like sponges and thereby interrupt miRNAs from interacting with their target mRNAs.

**FIGURE 1:**
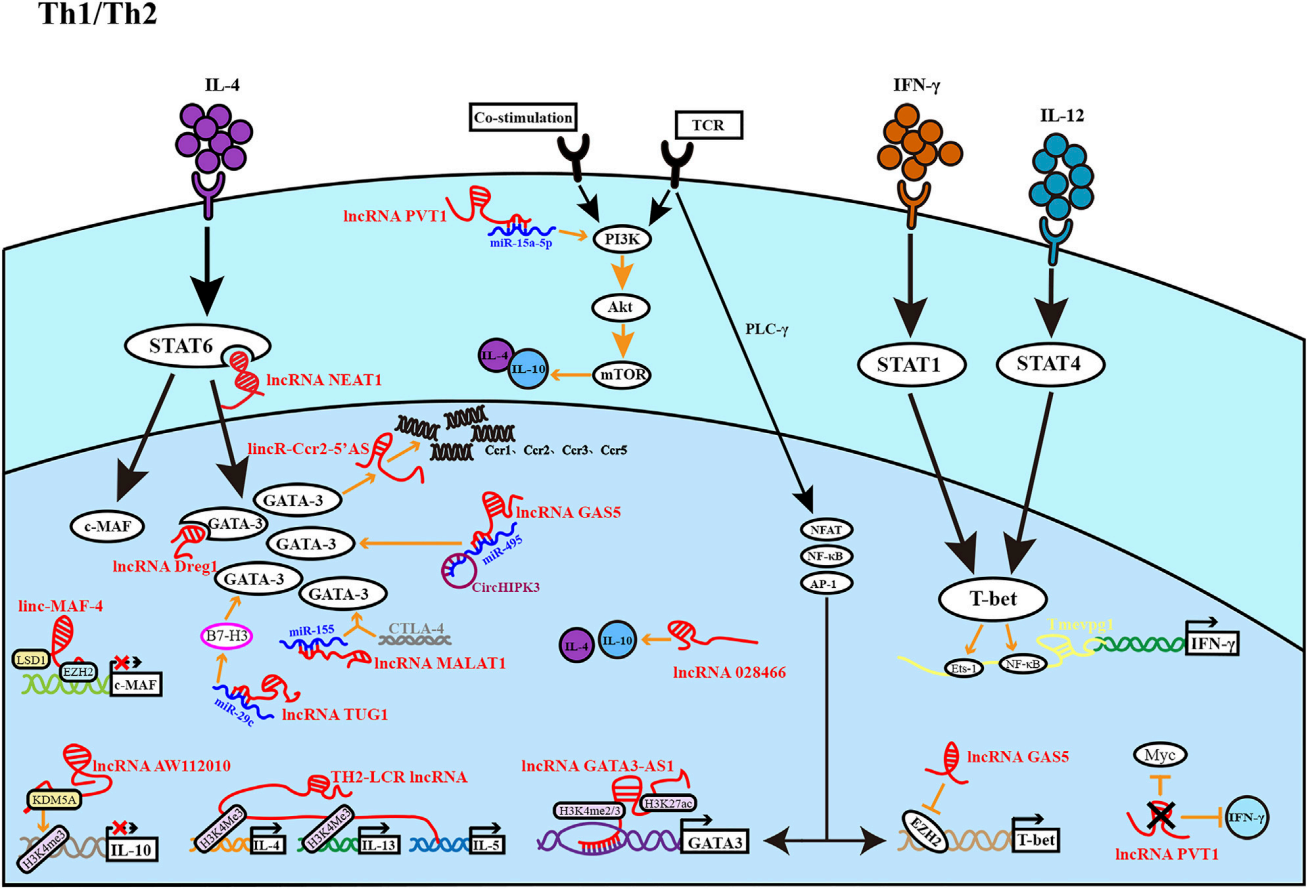
The figure depicts the role of lncRNA in the development of Th1 and Th2 cells. Th1 cells are regulated by light yellow lncRNA, and Th2 cells are regulated by red lncRNA. Most lncRNAs affect Th1 and Th2 differentiation at different stages to promote or inhibit cell differentiation.

LncRNA *FOXD3-AS1* was found to be downregulated in nasal mucosa of patients with AR, while IL-25, which promotes Th2-cell differentiation, was upregulated. Overexpression of the lncRNA *FOXD3-AS1* inhibits IL-25 expression in nasal epithelial cells and prevents the occurrence of the Th2 immune response (Zhang et al., 2020). LncRNA *FOXD3-AS1* has the potential to serve as a therapeutic target for AR. Additionally, the lncRNA *Dreg1* is intimately linked to the expression of GATA3 during Th2-cell differentiation and participates in the establishment of GATA3 expression (Chan et al., 2021), suggesting that *Dreg1* may promote Th2-cell differentiation. The expression of *LncRNA028466* decreased after inoculation with recombinant Echinococcus granulosus antigen P29 vaccine, and studies have shown that *LncRNA028466* can promote Th2-cell differentiation and the secretion of IL-10 and IL-4, but inhibits the secretion of Th1-related cytokines, IFN-γ and IL-2 (Wang et al., 2021). Cystic echinococcosis is a chronic parasitic disease for which there is still no commercial vaccine due to the complex host-parasite relationship. However, some effective antigens have been used as candidate vaccines for cystic echinococcosis prevention, such as recombinant Echinococcus granulosus antigen P29 (Wang et al., 2021). *LncRNA028466* may be involved in recombinant Echinococcus granulosus antigen P29 vaccination-mediated immune response by regulating the differentiation of CD4^+^ T cells during echinococcus granulosus infection (Wang et al., 2021). Finally, various knockdown and overexpression experiments indicate that lncRNAs play a key role in CD4^+^ T-cell differentiation. However, the characterization of these phenomena requires further study of the mechanisms by which they affect CD4^+^ T-cell differentiation (Figure 1).

## Th17 Lineage-Specific lncRNAs

Th17 cells are defined by their ability to produce the cytokine IL-17 as well as express the transcription factor RAR-related orphan receptor γ (RORγt). IL-6 and transforming growth factor (TGF)-β regulate RORγt expression via STAT3 and Smad proteins, respectively, promoting differentiation into the Th17 lineage (Chang et al., 2020). IL-21 also activates STAT3 as a surrogate cytokine. Alternatively, the STAT3 pathway can also be activated by IL-23, promoting Th17 lineage maintenance, but not commitment (Zhang, 2018).

The lncRNA DNA-damage-inducible transcript 4 (*lncDDIT4*) represses Th17-cell differentiation through the targeting effect of DDIT4, a cytoplasmic protein that is transcriptionally upregulated in the backdrop of DNA damage and is important for T cell survival and proliferation (Reuschel et al., 2015). DDIT4, located upstream of *lncDDIT4*, is a candidate target for *cis* regulation of *lncDDIT4*. The PI3K-AKT-mTORC1-S6K axis has been reported to actively regulate Th17 differentiation (Kurebayashi et al., 2012). Furthermore, *lncDDIT4* targets DDIT4, which activates the tuberous sclerosis complex 1/2 complex to inhibit the mTOR pathway, ultimately inhibiting IL-17 production (Zhang et al., 2018). The lncRNA *1700040D17Rik* was obtained from a mouse spleen cDNA library prepared from experimental autoimmune encephalomyelitis (EAE) model mice, and its expression is thought to affect Th17-cell differentiation. EAE mouse model is an ideal animal model for human multiple sclerosis (MS). MS is an inflammatory demyelinating disease of the central nervous system, the pathogenesis of which is associated with increased Th1 and Th17 lineages (Moser et al., 2020). Moreover, *1700040D17Rik* expression has been shown to be markedly decreased in EAE model mice but upregulated in mice treated with RHIL23R-CHR, an antagonist of endogenous IL-23, which functions to maintain Th17-cell differentiation. Functional studies have shown that *1700040D17Rik* is located near the *RORγt* gene and may affect the expression of *RORγt* in *cis*-mode and inhibit Th17-cell differentiation (Guo et al., 2017). LncRNA *1700040D17Rik* may be a potential therapeutic target or biomarker for MS.

*NEAT1* is also involved in Th17-cell differentiation. Rheumatoid arthritis (RA) is a chronic autoimmune disease dominated by inflammatory synovitis, and the increase of Th17 cells is closely related to RA. Therefore, inhibition of Th17 cell differentiation as a therapeutic target may be a potential strategy for RA (Shui et al., 2019). Studies have shown that *NEAT1* expression is upregulated in PBMC of RA patients and *in vitro* induced Th17 cells. By knocking down *NEAT1*, the arthritic degree of type II collagen induced arthritis in mice was alleviated. Binding of *NEAT1* to STAT3 prevents ubiquitination-mediated protein degradation and positively regulates STAT3 levels, thereby facilitating the regulation of Th17-cell differentiation. However, *NEAT1* knockdown alone suppresses Th17-cell differentiation, whereas simultaneous *NEAT1* knockdown and overexpression of STAT3 showed opposite effects; these findings reveal that *NEAT1* can be a helper molecule rather than a decisive factor in Th17 differentiation (Shui et al., 2019). As described above, lncRNA *GAS5* promotes Th2-cell differentiation. Studies have found that *GAS5* also plays a role in Th17-cell differentiation and is linked to the occurrence and progression of autoimmune disorders (Wu et al., 2019). Immune thrombocytopenia (ITP) is a common autoimmune disease in which increased differentiation of Th17 cells accelerates ITP progression (Li et al., 2020). *GAS5* expression is downregulated in PMBC of ITP patients and spleen tissues of ITP mice, while overexpression of *GAS5* in CD4^+^ T cells results in downregulated RORγt protein expression and suppresses IL-17 secretion. *GAS5* inhibits Th17 differentiation by promoting STAT3 ubiquitination, alleviates ITP, and provides a possible therapeutic target for ITP (Li et al., 2020). Similarly, the lincRNA *XLOC000261*, which is associated with Crohn’s disease, appears to negatively regulate RORγt protein expression (Braga-Neto et al., 2020). The functions of lncRNAs to bind transcription factors or regulate transcription as enhancer RNA are reflected in the above. We discovered that the same lncRNA could specifically transcription in different environments and participate in different signaling pathways, thus leading to the differentiation of CD4^+^ T cells in the direction of promoting disease, through *NEAT1* promoting of Th2 and Th17 cell differentiation in SLE and RA, respectively. *GAS5* is unique in that its expression is downregulated in ITP but upregulated in AR. In diverse conditions, an lncRNA can be specifically transcribed or repressed, facilitating disease progression.

LncRNA Opa-interacting protein 5 antisense transcript 1 (*OIP5-AS1*) was increased in peripheral blood lymphocytes of MS patients. By targeting *miR-140-5p*, lncRNA *OIP5-AS1* stimulates the RhoA/Rho kinase 2 (ROCK2) pathway, boosting Th17 subsets and accelerating EAE development *in vivo*. RhoA, a component of the Rho GTPase family, and its downstream factor ROCK2 are negatively modulated by *miR-140-5p*. Moreover, *miR-140-5p* inversely regulates the Th17 cell ratio and IL-17A expression, whereas *OIP5-AS1* reverses this effect (Liu R. et al., 2021). The role of *OIP5-AS1* in the development of MS may provide some candidate targets for the diagnosis and treatment of MS. LncRNA *Gm15575* accumulates specifically in Th17 cells and spleen cells of mice with EAE, through sponging *miR-686*, *Gm15575* promotes chemokine C-C motif chemokine ligand 7 (CCL7) expression (Bian et al., 2020). CCL7, a constituent of the chemokine C-C family, stimulates Th cells to produce cytokines and positively regulates Th17-cell differentiation; accordingly, the lncRNA *Gm15575* works on the pathogenesis of EAE (Bian et al., 2020). Inflammatory bowel disease (IBD) is a chronic autoimmune disease in which the imbalance between Th17/Treg cells is one of the causes. Th17 cells promote tissue inflammation, and Treg cells inhibit autoimmunity in IBD (Yan et al., 2020). The lncRNA intersectin 1–2 (*Lnc-ITSN1-2*) is strongly expressed in the intestinal mucosa and PBMCs of patients with IBD and has been shown to actively regulate IL-23R expression by sponging *miR-125a* (Nie and Zhao, 2020). Previous studies have shown that IL-23R can regulate the Janus kinase (JAK)/STAT pathway (Floss et al., 2015; Hummel et al., 2017) and the proliferation and differentiation of Th1 and Th17 cells is enhanced by IL-23R through regulation of JAK2/STAT3 and nuclear factor (NF)-κB pathways (Nie and Zhao, 2020). *Lnc-ITSN1-2* may provide a new perspective for the pathogenesis of IBD. Finally, lncRNA is important for Th cell function in both homeostasis and disease. LncRNAs can serve as guides for proteins to be recruited at specific DNA sites in both *cis* and *trans*. LncRNAs, on the other hand, may act as decoys, preventing proteins from contacting DNA. The diversity of lncRNAs functions determines that they may act on many network nodes of CD4^+^ T-cell differentiation, and they are present before, during and after transcription.

**FIGURE 2:**
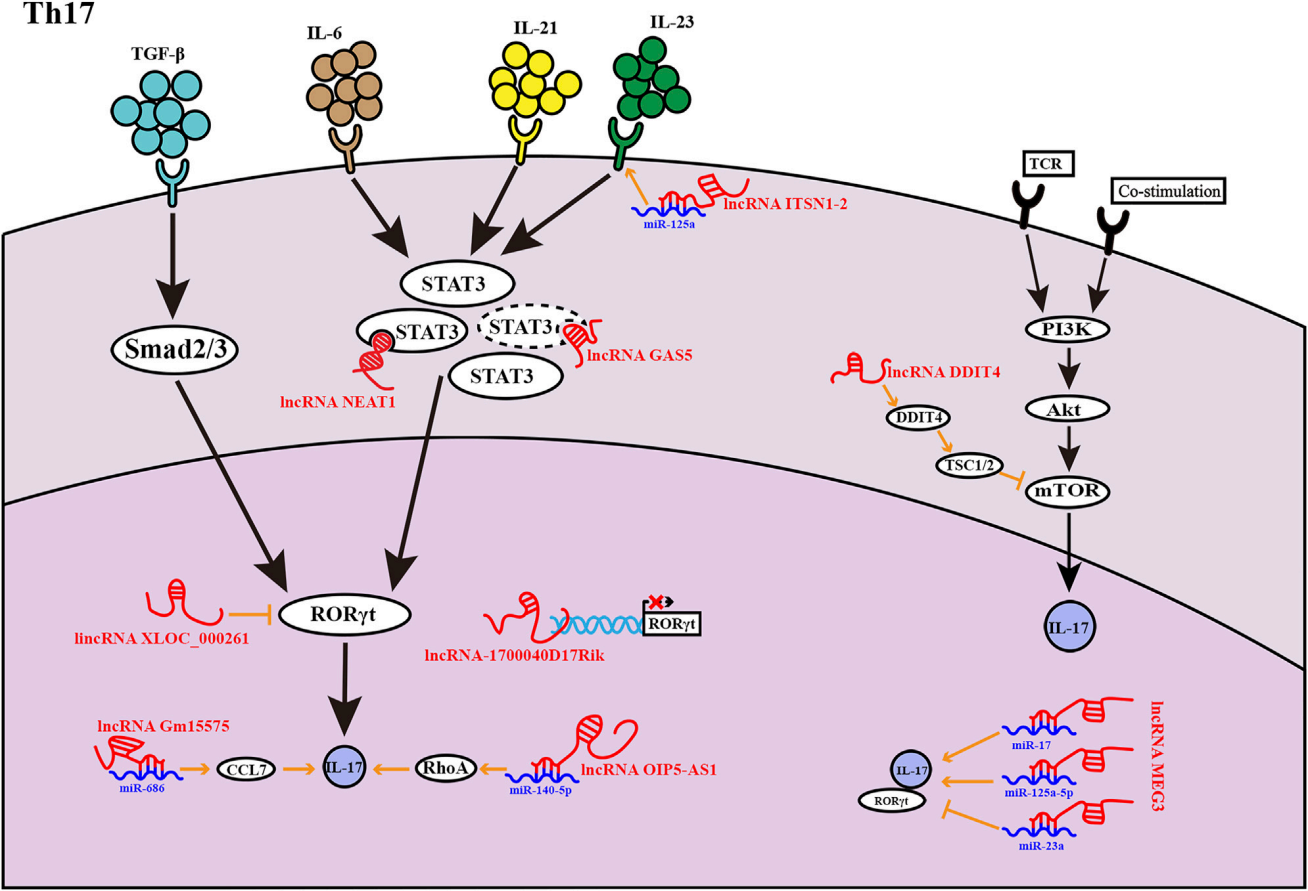
LncRNAs found in Th17 cells at various phases of development. LncRNAs influence Th17 cell development in a variety of ways. The lncRNA *GAS5* inhibits the transcription factor STAT3 expression, as indicated by the dashed lines.

Knockdown of *Lnc-DC*, which exists only in conventional human dendritic cells (DCs), inhibits DC differentiation and decreases the capacity of DCs to activate T cells. Moreover, *Lnc-DC* can bind to STAT3 and activate transcription (Wang et al., 2014). Because STAT3 is an important transcription factor in the Th17 lineage, we hypothesized that *lnc-DC* may be involved in Th17-cell differentiation (Figure 2).

## Role of lncRNAs in Treg Differentiation

CD25 and Foxp3, a genealogy-specific transcription factor, are markers of Tregs. TGF-β drives the downstream transcription factors Smad2 and Smad3 to promote Foxp3 transcription (Sekiya et al., 2016). Additionally, IL-2 promotes Treg production through STAT5 and co-induces Foxp3 expression with TGF-β (Josefowicz et al., 2012). In addition, TGF-β also regulates the balance between Th17 cells and Tregs (Konkel et al., 2017; Liu et al., 2018).

**FIGURE 3:**
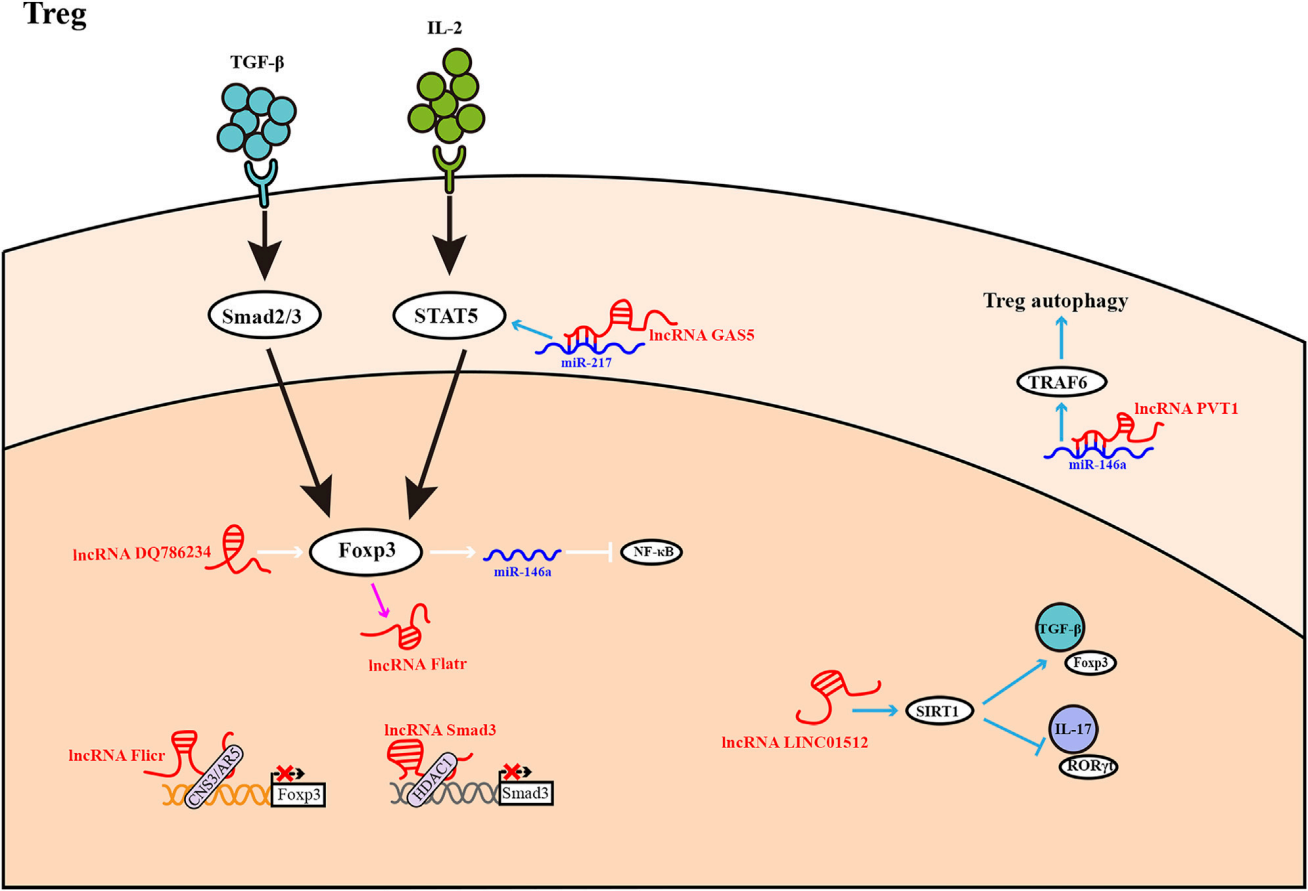
Treg differentiation is influenced by LncRNAs. Most lncRNAs operate as activators or inhibitors at different stages of Treg differentiation. The expression of lncRNA *Flatr* is dependent on Foxp3, but has no significant effect on Foxp3 expression.

The lncRNA Foxp3-specific lncRNA anticipatory of Tregs (*Flatr*), a member of the core Treg lncRNA transcriptome, is highly conserved and aggregated in sensitized Tregs. *Flatr* is expressed earlier than Foxp3, existing upstream of Foxp3, but depends on Foxp3 after its initial expression. In mice, defects in the *Flatr* gene result in minor Treg-induced damage. Accordingly, *Flatr* may have applications as a biomarker but does not appear to have a major functional role in mediating Foxp3 expression (Brajic et al., 2018). Oral lichen planus (OLP) is one of the common chronic inflammatory oral mucosal diseases, and Th17 response plays an important role in this disease (Monteiro et al., 2015). The lncRNA *DQ786243* is expressed increasingly in peripheral blood CD4^+^ T cells of OLP patients and is positively correlated with Foxp3 expression. Although the number of Tregs in OLP patients is higher than in normal subjects, their activity appears to be reduced. Overexpression of *DQ786243* was found to regulate *miR-146a* through Foxp3, *miR-146a* subsequently inhibits NF-κB signaling by blocking IL1R-associated kinase expression and tumor necrosis factor receptor-associated factor (TRAF) 6, increasing the proportion and inhibitory function of Tregs in patients with OLP (Wang et al., 2019b). LncRNA *DQ786243* may provide a possible treatment pathway for OLP. Tregs not only regulate immune tolerance but also play vital roles in transplantation immunity. In the event of the rejection period, the expression of *PVT1* is downregulated in Tregs and is inversely associated with *miR-146a* expression. In Tregs overexpressing *PVT1*, the expression of *miR-146a* is inhibited; this leads to upregulation of TRAF6, thereby enhancing the autophagy and inhibitory functions of Tregs and contributing to alleviation of transplant rejection (Lu et al., 2021). *PVT1* may be a novel therapeutic strategy for transplant rejection. The lncRNA Foxp3 intergene noncoding RNA (*Flicr*) is located adjacent to Foxp3, expressed specifically in mature Tregs and negatively regulated Foxp3 expression. *Flicr* regulates Foxp3 expression by modifying chromatin accessibility at the conserved noncoding sequence 3/accessible region 5 region of Foxp3 (Zemmour et al., 2017). The influence of *Flicr* is especially evident when IL-2 is in deficiency. It can also be shown that IL-2 contributes to the phenotypic stability of Tregs (Zemmour et al., 2017). Therefore, *Flicr* impacts Treg differentiation by reducing Foxp3 expression and may therefore also reduce the phenotypic stability of Tregs. Like many lncRNAs, *Flicr* affects autoimmune diseases through subtle molecular mechanisms (Sawant and Vignali, 2014). *LncRNA-Smad3* inhibits the differentiation of Tregs by mediating the expression of Smad3, the downstream signal transduction molecule of TGF-β. Notably, histone deacetylase 1 (HDAC1) combines with the promoter region of the *Smad3* gene to silence *Smad3* transcription. *LncRNA-Smad3* inhibits the expression of Smad3 by recruiting HDAC1, thereby affecting Treg differentiation (Xia et al., 2017). As a result, subtle changes in lncRNA expression levels may alter the Treg gene network, which cause the shift to inflammatory cell differentiation. In other words, lncRNA has a wide range of control over the growth and function of CD4^+^ T cells. LncRNAs are structurally plastic and fold into complex structures to bind to DNA, RNA, and proteins. Furthermore, lncRNAs may have some advantages because they do not require protein translation and respond faster (Figure 3).

## LncRNAs Modulate the Treg/Th17 Balance

TGF-β promotes the differentiation of naïve T cells into Tregs, whereas TGF-β induces the differentiation of Th17 cells when combined with pro-inflammatory factors such as IL-6 and IL-21. The dynamic balance of Tregs and Th17 cells is an important mechanism that functions to maintain immune homeostasis.

LncRNA maternally expressed gene 3 (*MEG3*) has been reported to be lost or reduced in many human tumors, and *MEG3* changes in various cancers suggest that it has the possibility to diagnose and evaluate the prognosis of cancer (Al-Rugeebah et al., 2019). One lncRNA may play roles in Th17 divergence through absorbing distinct miRNAs in various disease models. LncRNA *MEG3* is highly expressed in CD4^+^ T cells of ITP patients, resulting in an imbalance in the Treg/Th17 ratio through sponging *miR-125a-5p*; this then promotes Th17-cell differentiation and causes an immune imbalance in ITP patients (Li et al., 2016). *MEG3* may be a target for restoring Th17/Treg immune balance (Wang et al., 2019a). Aplastic anemia (AA) is a life-threatening autoimmune disease, in which the main mechanism is hematopoietic tissue damage caused by T cell hyperfunctio (Wang et al., 2019a). The expression of *MEG3* in CD4^+^ T cells in AA patients is much lower than in normal individuals, although the expression of *miR-23a* is significantly higher. Importantly, low *MEG3* expression leads to excessive CD4^+^ T-cell differentiation into Th1 and Th17 cells, which significantly increases the levels of RORγt and T-bet and IL-17a and enhances the levels of IL-17a and IFN-γ, thereby exacerbating the symptoms of AA (Wang et al., 2019a). *MEG3* overexpression reverses this effect by absorbing *miR-23a* (Wang et al., 2019a). LncRNA *MEG3* also has some roles in asthma. The expression level of *MEG3* in CD4^+^ T cells in peripheral blood of asthmatic patients increased, while the *miR-17* level decreased. *MEG3* upregulates RORγt through sponge *miR-17*, tilting Treg/Th17 balance toward Th17 in asthmatic patients (Qiu et al., 2019). *MEG3* may also be a potential therapeutic target for immune-related diseases in the future.

Necrotizing enterocolitis (NEC) is a severe inflammatory bowel necrosis that occurs mainly in premature infants. The imbalance of Th17/Treg is increasingly associated with NEC (Gao et al., 2021). The lncRNA *LINC01512* is downregulated in NEC patients, while melatonin upregulates *LINC01512* through adenosine monophosphate-activated protein kinase (AMPK) signaling and interacts with sirtiun 1 (SIRT1), a regulator of various cellular and bodily processes. Furthermore, *LINC01512* promotes Treg differentiation and inhibits Th17 differentiation by enhancing SIRT1 expression, thereby administering to correct the imbalance of Treg/Th17 ratio (Gao et al., 2021). The above melatonin-induced pathway may be a novel way to suppress inflammation.

Th17/Treg imbalance plays an important role in the pathogenesis of pneumonia (Chi et al., 2021). In children with pneumonia, *GAS5* expression is downregulated, and the proportion of Th17 cells increases. Conversely, overexpression of *GAS5* can correct the imbalance in Tregs/Th17 cells. Specifically, *GAS5* reduces the binding of *miR-217* with its target gene *STAT5* by sponging *miR-217*, leading to upregulation of STAT5 expression and promoting the differentiation of Tregs (Chi et al., 2021). This mechanism may provide insights into the development of novel approaches for treating pediatric pneumonia.

The lncRNA *MALAT1* is downregulated in the acute stage of EAE mice, similar to the trend observed for *MALAT1* expression in the brain tissues of patients with MS. Low expression of *MALAT1* leads to differentiation of CD4^+^ T cells into Th1 and Th17 cells, and increases the secretion of IFN-γ and IL-17, while reducing Treg differentiation (Masoumi et al., 2019). Although the molecular mechanism has not been reported, changes at the cellular level and cytokine level may also explain this phenomenon to some extent. LncRNA *MALAT1* may be a potential target for future therapeutic interventions in inflammatory demyelination. (Figure 2; Figure 3).

Many immune diseases are caused by a Th17/Treg imbalance. Although the differentiation process of Th17 and Treg cells is irreversible, the phenotype of CD4^+^ T cells can be controlled. LncRNA analysis provides compelling evidence to explain the plasticity of CD4^+^ T-cell development from a molecular perspective, which aids in the discovery of new therapeutic targets for immune diseases.

## LncRNAs Involved in Th9 and Tfh Cell Development

Th9, as a class of newly discovered CD4^+^ Th cells, expresses the transcription factor PU.1 and the cytokine IL-9 (Chen et al., 2019). Although no studies have found that lncRNAs participate in the differentiation of this subgroup, IL-9 and the lncRNA *Gm13568* have been shown to be correlated in MS and EAE (Liu X. et al., 2021). Therefore, lncRNAs may also be involved in Th9-cell differentiation, waiting for us to explore.

Tfh cells are also a relatively new Th cell type, and their differentiation mainly depends on the pedigree-defined transcription factor Bcl6 expression as well as the other important transcription factors Maf and STAT3 (STAT3 and STAT4 in humans) (Crotty, 2014; Vinuesa et al., 2016). One study demonstrated that the lncRNA *MALAT1* is overexpressed in T cell subsets but is not a critical regulator of T cells; furthermore, *MALAT1* has been shown to be nonessential for Tfh-cell differentiation (Yao et al., 2018). Although lncRNAs have not yet been shown to regulate Tfh-cell differentiation, relevant sequencing studies have indicated that many lncRNAs are relevant to Tfh pedigree transcription factors and cytokines (Deng et al., 2021; Zhang et al., 2021). Therefore, the roles and specific mechanisms of lncRNAs in Tfh-cell differentiation are waiting for further study.

## Conclusion and Perspective

Several RNA-based therapies have been developed, such as small interfering RNA (siRNA), antisense oligonucleotides (ASOs), and short hairpin RNA (shRNA). Currently, the US Food and Drug Administration and/or the European Medicines Agency have approved various RNA-based treatments. Fomivirsen was the first ASO to be approved to target the cytomegalovirus IE-2 mRNA (Stein and Castanotto, 2017). Patisiran was licensed in 2018 for the treatment of hereditary thyroxine amyloidosis, the first RNAi-based medication (Adams et al., 2018). In recent years, Givosiran, Viltolarsen, Inclisiran, and others have been approved (Winkle et al., 2021). Nedosiran for primary type 1 hyperoxaluria, primary type 2 hyperoxaluria, renal illness, and urinary disease, Alicaforsen for Crohn’s disease, and additional RNA treatments are in phase II or III clinical trials. However, no lncRNA-based therapy has been approved for use in humans. The variety of functions of lncRNAs indicates a variety of therapeutic targets. Natural antisense transcription (NAT), in which lncRNA is transcribed into coding genes in the antisense direction and negatively regulates them in the *cis*, is a promising path to pursue (Winkle et al., 2021). In the experimental stage, NAT has showed encouraging outcomes. “AntigoNATs” are ASOs that target NATs. In one investigation, upregulation of the *SCN1A* healthy allele using antagoNATs targeting SCN1ANAT alleviated Dravet syndrome (Hsiao et al., 2016). AntagoNATs were found to upregulate brain-derived neurotrophic factor, a protein linked to memory formation, in another study (Modarresi et al., 2012). These findings point to the potential of lncRNA-based treatments. Committing to researching the regulation mechanism of lncRNA may, to some extent, redefine the controller and regulator of Th-cell fate and provide a new direction for therapeutic intervention in immune diseases. Simultaneously, we must realize that genetic medications are difficult to administer precisely targeted and easy to provoke immune responses and other adverse effects, which is a severe challenge.

In conclusion, lncRNAs play an important role in CD4^+^ T-cell differentiation, revealing the complexities of CD4^+^ T-cell development and function at the molecular level. Because the specific molecular mechanism of some lncRNAs in the differentiation process of CD4^+^ T cells is unknown, we should continue to investigate their molecular mechanism and related undiscovered lncRNAs. Meanwhile, investigating the potential role of lncRNA in CD4^+^ T cells can help us better understand the role of the non-coding transcriptome in adaptive immunity. Thus, determining the pathways by which lncRNAs regulate CD4^+^ T-cell development and plasticity can help us better understand the immune response regulation mechanisms, as well as identify the causes of CD4^+^ T-cell functional alterations in illnesses and identify new therapeutic targets.
